# Deep Learning Applications in 12-lead Electrocardiogram and Echocardiogram

**DOI:** 10.31662/jmaj.2024-0195

**Published:** 2024-09-27

**Authors:** Masamitsu Nakayama, Ryuichiro Yagi, Shinichi Goto

**Affiliations:** 1 Division of Cardiovascular Medicine, Department of Medicine, Brigham and Women’s Hospital, Boston, USA; 2 Harvard Medical School, Boston, USA; 3 Division of General Internal Medicine & Family Medicine, Department of General and Acute Medicine, Tokai University School of Medicine, Isehara, Japan

**Keywords:** Electrocardiogram, Echocardiogram, Deep learning, Artificial intelligence, Convolutional neural network, Cardiovascular disease

## Abstract

Artificial intelligence (AI), empowered by advances in deep learning technology, has demonstrated its capabilities in the medical field to automate tedious tasks that are otherwise performed by humans or to detect or predict diseases with higher accuracy compared with experts. Given the ability to take complex multidimensional data as input, AI models have primarily been applied to complex medical imaging and time-series data. Another prominent strength of AI applications is its large scalability. The field of cardiovascular medicine uses various noninvasive and accessible metrics that produce a large amount of complex multidimensional data, such as electrocardiograms (ECGs) and echocardiograms. AI models can increase the utility of such modalities. Simple automation of conventional tasks using AI models provides significant opportunities for cost reduction and capacity expansion. The ability to improve disease detection or prediction at scale may provide novel opportunities for disease screening, enabling early intervention in asymptomatic patients. For example, AI-enabled pipelines can accurately identify cardiomyopathies and congenital heart diseases from a single ECG or echocardiogram recording. The detection of these diseases using the conventional approach usually requires complicated diagnostic strategies or expensive tests. Therefore, underdiagnosis is a huge problem. Using AI models to screen these diseases will provide opportunities for reducing missed cases. The utility of AI models in the medical field is not limited to the development of clinically useful models. Recent research has shown the promise of AI models in mechanism research by combining them with genetic and structural analyses. In this review, we provide an update on the current achievements of the innovative AI application for ECG and echocardiogram and provide insights into the future direction of AI in cardiovascular care and research settings.

## 1. Introduction

Cardiovascular diseases (CVD) are one of the leading causes of death worldwide ^(1), (2), (3)^. These factors significantly affect people’s health and longevity. Because proper management of CVD and the underlying lifestyle diseases can improve prognosis and reduce mortality owing to advances in cardiovascular care, early detection and prevention are of great importance. The 12-lead electrocardiogram (ECG) and echocardiogram have long been recognized as essential tools in cardiovascular care. ECG measures the electrical activity of the heart from the body surface, whereas echocardiogram allows real-time imaging to assess cardiac structure and function using ultrasound. Given their noninvasive, inexpensive, and accessible nature compared with other cardiovascular tests, such as magnetic resonance imaging (MRI) or catheterization procedures, both ECG and echocardiogram are widely performed in developed and developing countries. The number of tests is expected to continue rising; thus, ECG and echocardiogram will continue to play crucial roles in the diagnosis and management of CVD.

Although ECG and echocardiogram provide substantial information about cardiac abnormalities, interpreting the results remains challenging. Intensive training is required for professional evaluation, and even with such training, significant variability still persists both between different observers (interobserver variability) and within the same observer (intraobserver variability). Furthermore, recent studies on artificial intelligence (AI) models achieving better performance than experts indicate that some information contained in the data is not fully used even by those who have undergone intensive training.

Various imaging modalities are being used in the healthcare field, and to date, deep learning (DL)-based algorithms have shown promising performance in the analysis of diagnostic images, including X-rays ^(4)^, computed tomography ^(5)^, and MRI ^(6)^. Similarly, DL technique is broadly and successfully employed in cardiovascular research for complicated tasks beyond physician recognition. In light of these achievements, DL applications have started to be implemented in clinical settings ^(7)^. In this review, we discuss how DL applications are developed and implemented to improve diagnostic performance by analyzing ECG and echocardiogram data in cardiovascular medicine. Additionally, this review explores the current limitations and potential future directions of DL applications.

### 1-1. DL models

**Figure 1.**
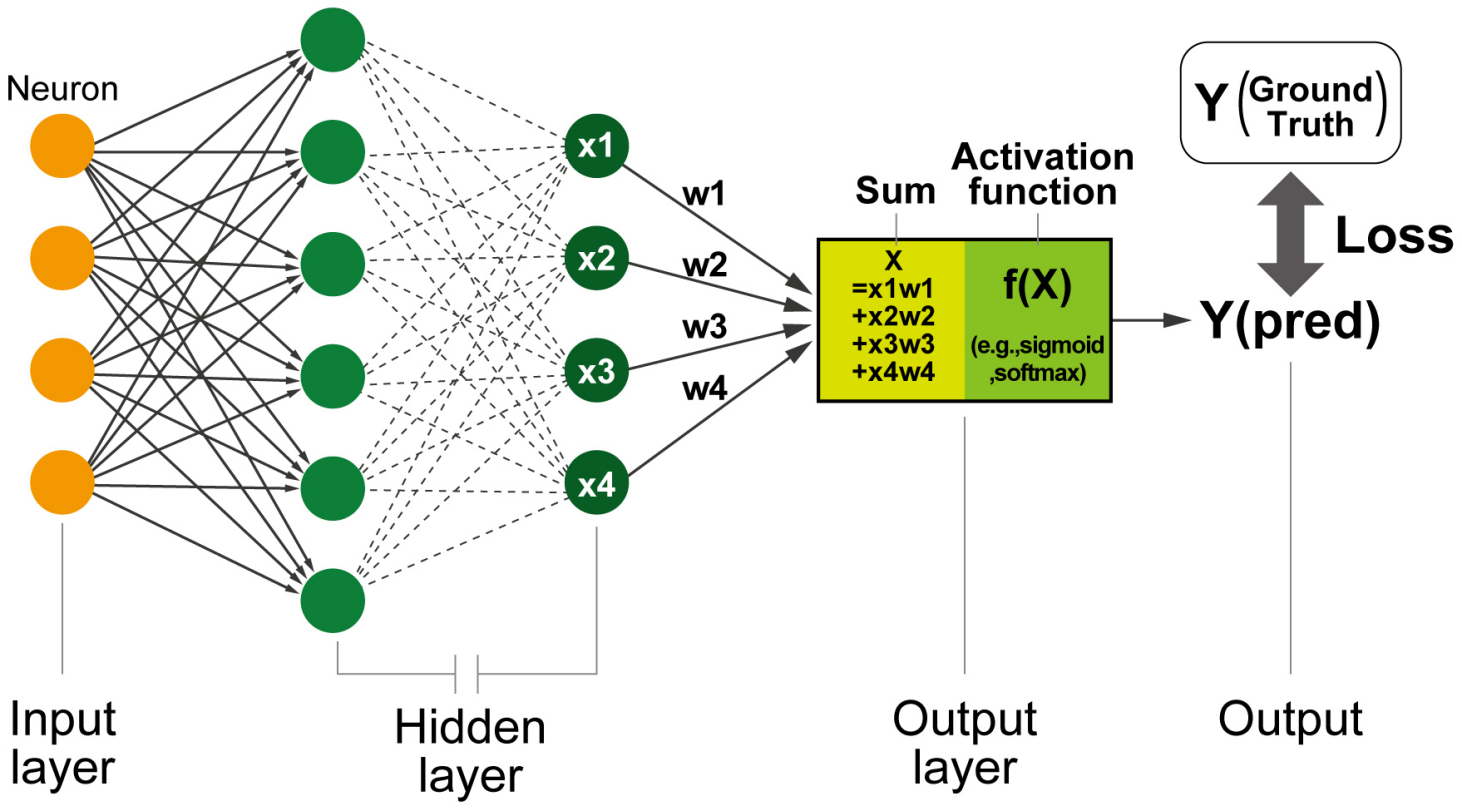
Schematic of the neural network architecture A neural network mimics the structure of human brain cells and comprises input, hidden, and output layers. The neuron, which is the basic unit of the neural network, contains adjustable parameters called weights. Each neuron calculates output values from the inputs and weights and then passes these individual outputs to the next neurons. In the final output layer, the network computes the aggregated values from the preceding neurons and applies an activation function (e.g., sigmoid for binary classification and softmax for multiple classifications) to generate the final output. The loss, which is defined as the difference between the predicted output and ground truth, is minimized during the training process. Neural networks can be stacked to greater depths, which allows them to approximate various functions and handle diverse input types.

Before diving into the details of DL applications on ECG and echocardiogram, it is essential to clarify the fundamental concepts of AI and DL. In the current medical field, “AI” refers to a statistical model, including machine learning and, more specifically, DL models. The term “deep” in DL refers to the stacking of numerous layers within neural network (NN) architectures in hidden layers, which enables the extraction of progressively more meaningful feature representations (Figure 1). Neurons, a unit of NNs that imitate the functioning of human neuronal cells, are connected to different neurons, receive inputs, and provide outputs to the next neurons. The parameters, called “weights,” are adjusted to minimize the difference between the output and the actual answer (also known as loss or error) using gradient-based optimization methods. This fundamental design of an NN is referred to as a “fully connected NN” or a “multilayer perceptron.” This architecture is capable of learning features from multidimensional data; however, it faces challenges when explicitly managing the spatial and temporal positions of the data. To address these limitations, specialized neuron units, such as convolutional NNs (CNNs) and recurrent NNs (RNNs), were developed for image and time-series data analyses ^(8)^. The RNNs return the output of the unit to an auxiliary input of the same unit. This can be understood as multiple units with rectified data transfers between the units next to them. This structure is sensitive to changes in the data order in the input. Therefore, the unit is useful in learning the order of data and is used for time-series data and languages. Conversely, CNNs use a series of filters, called “kernels,” that slide across the image to detect features relevant to a certain task. Through the training process, a unit of CNNs adjusts the weights of the kernels to minimize the difference between the outputs and the labels (ground truth). The dimensionality of these kernels can be changed to fit the input data, thereby allowing the processing of one-dimensional sequential, two-dimensional image, and three-dimensional video data. This extendibility has facilitated the direct analysis of ECG and echocardiogram data without significant manual preprocessing, resulting in the construction of models that maximally use the information content of intricate inputs. As a result, CNN-based DL models are now the most widely used DL systems for ECG and echocardiogram analyses.

## 2. DL Application to ECG

**Figure 2.**
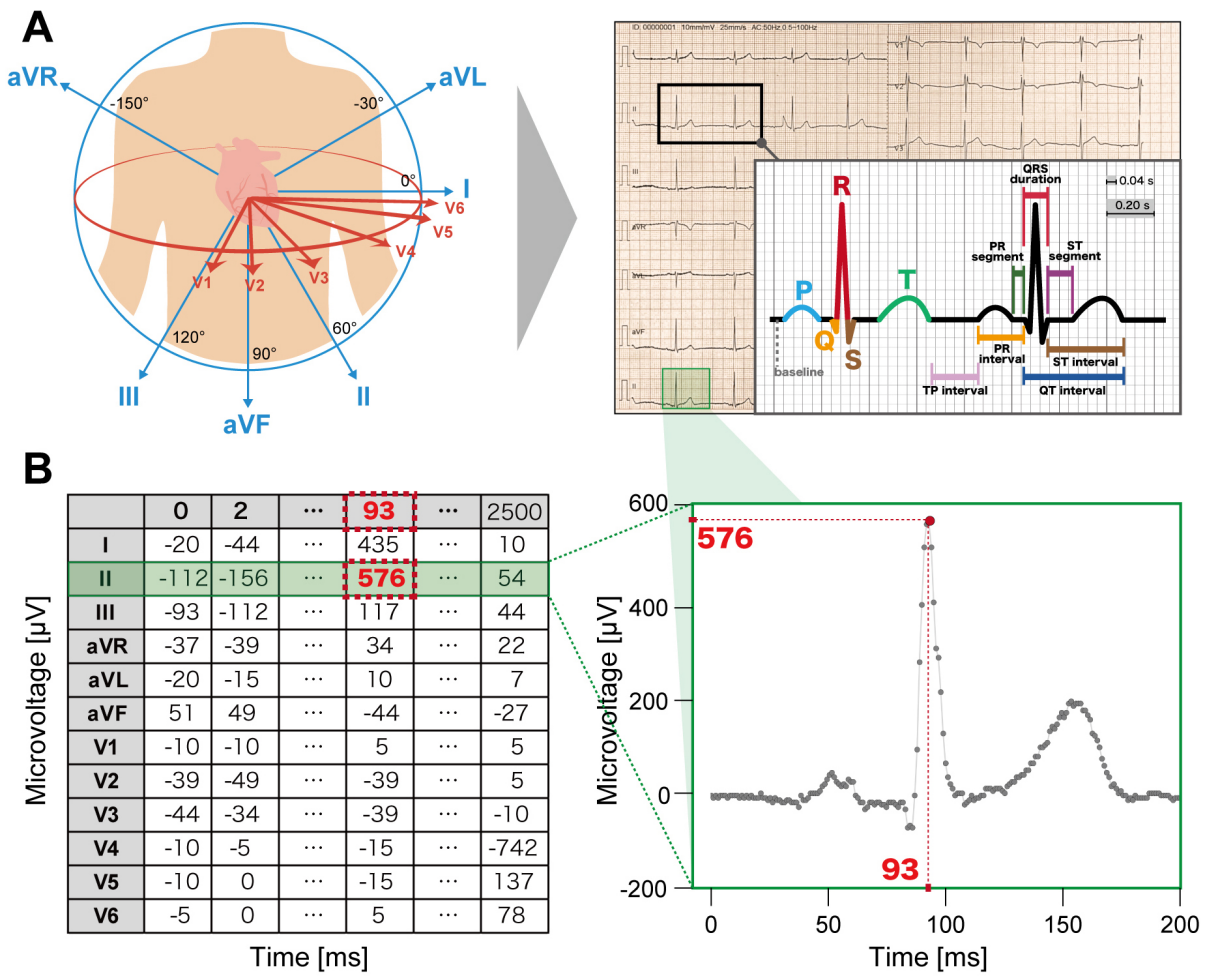
Overview of 12-lead electrocardiogram (ECG) signal data A. A common ECG records cardiac electrical activities from 12 different angles and directions over a 10-s period on the body surface. Each heartbeat is characterized by distinct components of the waveforms, and various measurements are derived. B. The recorded 12-lead ECG comprises numerical values corresponding to the amplitudes of the raw ECG signal. An example 2D matrix of 12-lead ECG is shown with units of microvolts versus milliseconds. ECG data typically consist of recordings from 12 leads over 10 s at either 250 or 500 Hz, represented as a 12 × 2500 or 12 × 5000 matrix.

Modern DL models are designed to input raw ECG voltage data (Figure 2). The raw voltage of ECG consists of time-series numerical values of voltage (mV) recorded each ms. Conventional ECG data are composed of 12 leads with a recording of 10 s each at either 250 or 500 Hz, commonly and instantly represented by a 12 × 2,500 or 5,000 matrices. Thus, the format of the raw ECG voltage data is considered well standardized and thus more suitable for DL analyses than for other modalities.

The application of DL models in ECG analysis has already been widely demonstrated. By leveraging a large amount of accumulated ECG signal data, such as those stored in electronic health records (EHRs), DL models have demonstrated the capability to perform various clinical tasks. There are mainly three types of applications of DL models to ECG that we will discuss in this paper. The first type mainly focuses on automating human tasks. The automatic classification of ECG abnormalities can help human experts read ECGs at large scales by enabling faster interpretation. The second type aims to train DL models to detect heart diseases by identifying multiple, subtle, nonlinear-related patterns in ECG that are often not recognizable by experts, and the third type attempts to use DL models to facilitate the prediction of progression and risk stratification of CVD by recognizing electrical abnormalities that proceed structural abnormalities and outcomes.

### 2-1. DL application to automate traditional ECG interpretation

The increasing demand for ECG interpretation has driven efforts toward automated and accurate ECG analysis. Primarily, ECG waveforms are plotted for visual analysis and categorized into >70 abnormalities based on their morphology defined by the deviation from the normal waveform ^(9)^. Although algorithm-based interpretation is widely used in ECG devices, it has limitations that may affect medical decision-making ^(10)^. Considering its compatibility with ECG data, DL-based techniques are expected to demonstrate better performance than commercial ECG software based on conventional process techniques. Several studies have revealed that DL models can outperform physicians in classification tasks. After learning sufficient ECG recordings obtained from the EHR data, CNN-based models were capable of categorizing ECG abnormalities with accuracy equivalent to that of expert cardiologists ^(11), (12)^. Because the interpretation accuracy of ECG varies across training levels ^(13)^, this DL application can help improve conventional ECG interpretation and reduce the resources required for ECG reading.

### 2-2. DL application for CVD detection

DL applications show great promise for detecting CVD, which is often not recognizable by experts from ECG. In 2019, the DL application enabled the identification of patients in need of emergency catheterization ^(14)^ and those with impaired left ventricular ejection fraction (LVEF) ^(15)^ from a single recording of ECG signals. Goto et al. ^(14)^ developed a long short-term memory model for ECG performed in the emergency department to predict the need of catheterization procedure with an area under the receiver operating curve (AUROC) of 0.88. Attia et al. ^(15)^ demonstrated that a DL model trained with >50,000 ECG signal data could detect patients with left ventricular systolic dysfunction (defined as echocardiographically observed LVEF <35%) with high accuracy (AUROC 0.93). Following these achievements, this novel approach, by leveraging the combination of DL and ECG, has been applied for various tasks that are difficult for human physicians, including the detection of paroxysmal atrial fibrillation from sinus rhythm ECG ^(16)^, ischemic heart disease ^(17)^, valvular diseases ^(18), (19)^, electrolyte abnormalities ^(20), (21)^, and pulmonary artery hypertension ^(22), (23)^. Interestingly, DL algorithms were shown to be capable of detecting cardiomyopathies and congenital heart diseases, which often require complex diagnostic approaches. Goto et al. developed DL-enabled pipelines that can accurately detect cardiac amyloidosis ^(24)^ and hypertrophic cardiomyopathy (HCM) ^(25)^. Similarly, a CNN-based robust algorithm was successfully constructed to identify atrial septal defect ^(26)^. These DL algorithms performed robustly against patient backgrounds and significantly outperformed expert cardiologists whose readings were based on conventional ECG waveform interpretations. Importantly, these models were validated on external datasets that were independent of the training data, strongly supporting their high generalizability ^(24), (25), (26), (27)^. It is noteworthy that some models were validated in a prospective manner ^(28), (29)^. These findings indicate the readiness of DL application for having a favorable effect on the clinical settings.

### 2-3. DL application for predicting cardiovascular outcomes

In addition to the aforementioned achievements, DL models have demonstrated their ability to predict hidden diseases or statuses before any mechanical or structural abnormalities become observable from other modalities. In other words, the output of DL models can function as a new digital biomarker that can contribute to accurate disease prediction and risk stratification. For instance, a CNN-based model can accurately estimate chronological age from ECG (R^2^ = 0.837) ^(30)^, and the difference between “heart-age” calculated by a DL model analyzing ECG recordings and chronological age was shown to serve as a strong prognostic factor ^(31), (32)^.

Multiple studies have shown the potential of DL approach for specific purposes. Ouyang et al. ^(33)^ developed a DL system to predict clinical outcomes in patients undergoing medical procedures using preoperative ECG testing within 30 days before the procedures. DL-enhanced ECG analyses were also performed for predicting 1-year ^(34)^ and in-hospital ^(35)^ mortality. Yagi et al. ^(36)^ developed a CNN-based model to predict cancer therapy-related cardiac dysfunction induced by anthracycline using baseline ECG data obtained before the initiation of chemotherapy using a transfer learning approach. These findings show that the DL application may hold promise even as a prognostic indicator affecting the clinical decision-making process.

## 3. DL Application to Echocardiograms

Echocardiogram is a unique imaging modality that allows real-time cardiac assessment, providing a wealth of information by capturing the heart’s movements from different views and angles. Accurate evaluation of cardiac structure and function, such as wall motion, chamber size, and valve condition, helps in clinical diagnosis and guiding the best treatment options for patients with CVD. However, the variability generated by manual operations imposes a significant burden on accurate assessment ^(37)^. Additionally, the echocardiogram interpretations are influenced by the individual examiners’ experience. Thus, interobserver and intraobserver variability in echocardiogram readings remains a significant challenge in current clinical workflows.

**Figure 3.**
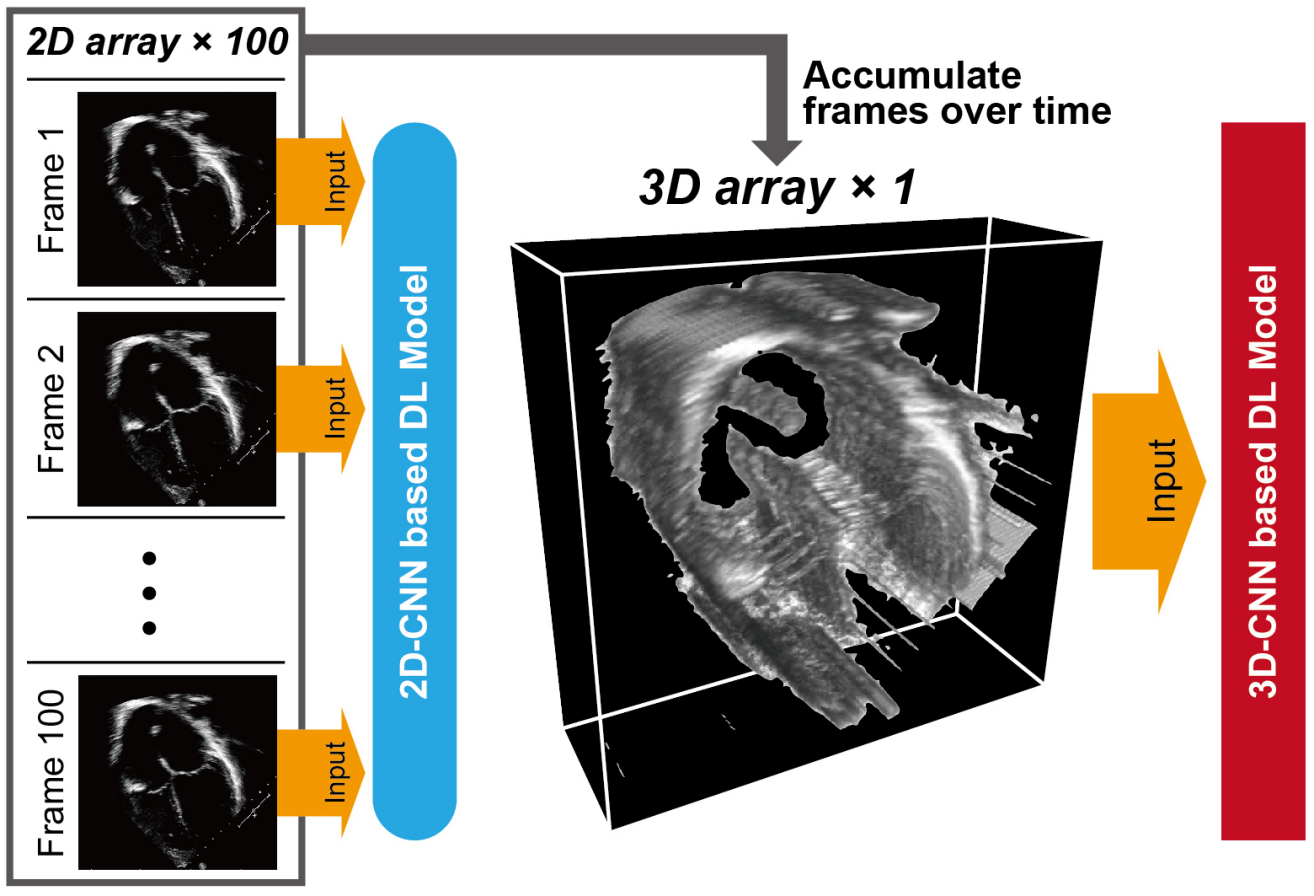
Comparison of echocardiogram video data as 2D and 3D arrays as inputs for DL models The echocardiogram video is a series of frame images stacked in the time direction. While inputting frame images as a 2D array into a DL model loses the dynamic information along the time axis, a 3D array, which stacks frame images, retains both temporal and spatial dynamics as a 3D shape. DL, deep learning; CNN, convolutional neural network.

Analogous to ECG, DL models are capable of recognizing various patterns in echocardiogram data. The early DL model pipelines were designed to use echocardiogram data as individual image frames split from sequential video data. The concept of frame-based DL models was applied to various tasks, including view classification, image segmentation, measurements of cardiac structure and function, and disease detection ^(38), (39), (40)^. Despite the potential of frame-based DL models to automate human tasks, a significant limitation is their inability to retain dynamic information in echocardiogram videos when analyzed as individual frames. More recent DL algorithms have attempted to learn distinctive features across temporal and spatial dimensions by treating the temporal axis as the third dimension. This approach enabled the DL models to use dynamic information as a three-dimensional “shape” (Figure 3) and achieved accurate measurements, abnormality detection, and diagnosis.

### 3-1. DL application to automate traditional echocardiogram assessment

DL applications have demonstrated the ability to assess various measurements traditionally performed by human sonographers. Ouyang et al. ^(41)^ developed a video-based DL model for semantic segmentation of the left ventricle and estimation of LVEF values from echocardiogram videos (R^2^ of 0.81 compared with LVEF values calculated by human experts). This approach is applicable for the automated assessment of LVEF in pediatric patients ^(42)^. Furthermore, DL technique was successfully applied to quantify other structural and functional metrics, such as left ventricular structure ^(43)^ and global longitudinal strain ^(44)^. It is also noteworthy that the DL-based algorithm for calculating LVEF was tested in a randomized trial ^(45)^, concluding that LVEF assessment by DL algorithm was noninferior to assessment by sonographers. These findings highlight the potential utility of DL-enhanced echocardiography in hospital settings.

### 3-2. DL application for disease identification

DL applications may help diagnose CVD beyond human capability using dynamic echocardiographic information. The first DL model published in this category, taking echocardiogram data as input used dynamic information to detect cardiac amyloidosis ^(24)^. Their model yielded AUROCs of 0.89–1.00 across five institutions. Similarly, researchers applied the DL-based technique to detect HCM, with AUROCs of 0.90–0.92 in its detection across four institutions ^(25)^. This dynamic information approach also enabled the accurate detection of valvular diseases, such as aortic stenosis (AS) ^(46)^.

DL-enhanced echocardiogram models can also be used as digital biomarkers. A CNN-based model was trained to assess the risk of progression of AS using single-view long-axis echocardiogram videos without Doppler characterization ^(47)^. DL algorithms using echocardiography were also applied to estimate coronary artery calcification score ^(48)^ and predicting short- to long-term mortality ^(49)^. A recent advancement in this field involves the use of a vision–language foundation model that can process echocardiogram video and text data simultaneously. This model enhanced interpretation by aligning relevant textual information with critical regions of interest in the images, such as implanted intracardiac devices ^(50)^. These advancements support the strength of DL-enabled echocardiographic approach for detecting subtle indications of diseases, helping to prevent the onset of future CVD events.

## 4. Current Challenges and Future Direction

Although DL models have demonstrated promise in their application to various clinical tasks in cardiovascular fields, several limitations still need to be addressed before their broad implementation in clinical settings. The first problem is the interpretability of DL models, which is a challenge commonly referred to as the “black box.” The internal training process, which automatically calculates weights without specific instructions from researchers, makes it challenging for humans to understand how the model handles information and draws conclusions. Although some techniques such as gradient-weighted class activation mapping could visualize which parts of the input data the model focuses on ^(18), (19), (51)^, it remains unclear “how and why” DL model processes this information. Until a novel technology addresses this problem, the applications of DL models will be limited to situations in which interpretability is not strongly required.

**Figure 4.**
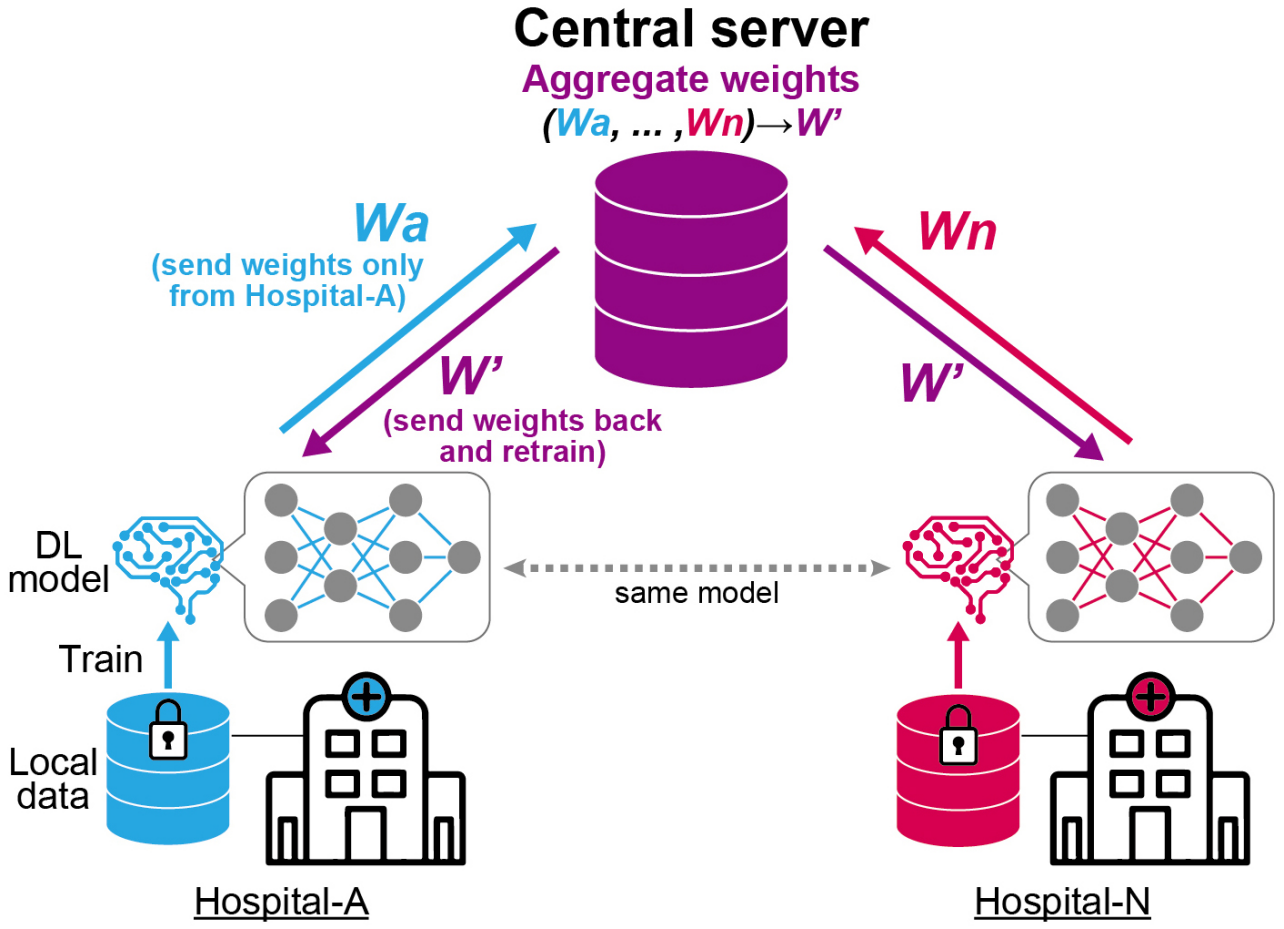
Schematic of federated learning The proposed method trains a deep learning (DL) model without data sharing. The same model architectures are distributed to multiple institutions for training on local data. The trained weights, which are no longer interpretable, are sent to a central server for aggregation. The central server then sends the aggregated weights back to each institution for further training.

The second problem is the generalizability of the model across various healthcare settings. The performance of DL models can be affected by various factors, such as variations in patient populations, imaging equipment, and acquisition protocols. It has been reported that many DL algorithms have diminished performance on external datasets with no clear explanations ^(52), (53)^. Importantly, the models should be validated on external cohorts that have similar characteristics to the populations where the models should be deployed. Although a previous study indicated the utility of ECG for a CVD screening purpose in general population ^(54)^, most of DL-enabled models were validated in hospital settings, where participants’ conditions are generally more severe. Increasing the variability of the data used for training by collecting data from multiple institutions is one way to improve generalizability. However, despite the growing need to collect large quantities of high-quality data from multiple institutions, data sharing across multiple locations can pose significant privacy and procedural barriers. To address this issue, federated learning is considered a practical approach (Figure 4) ^(25)^. This technique facilitates model training by transferring the weights of the model across multiple institutions without sharing the clinical data. The output from each local node, which is trained on local clinical data, is sent to the central server. The central server aggregates these outputs into a single model without accessing the original clinical data. This approach may help address the difficulty of collecting clinical data across institutions due to privacy concerns.

**Figure 5.**
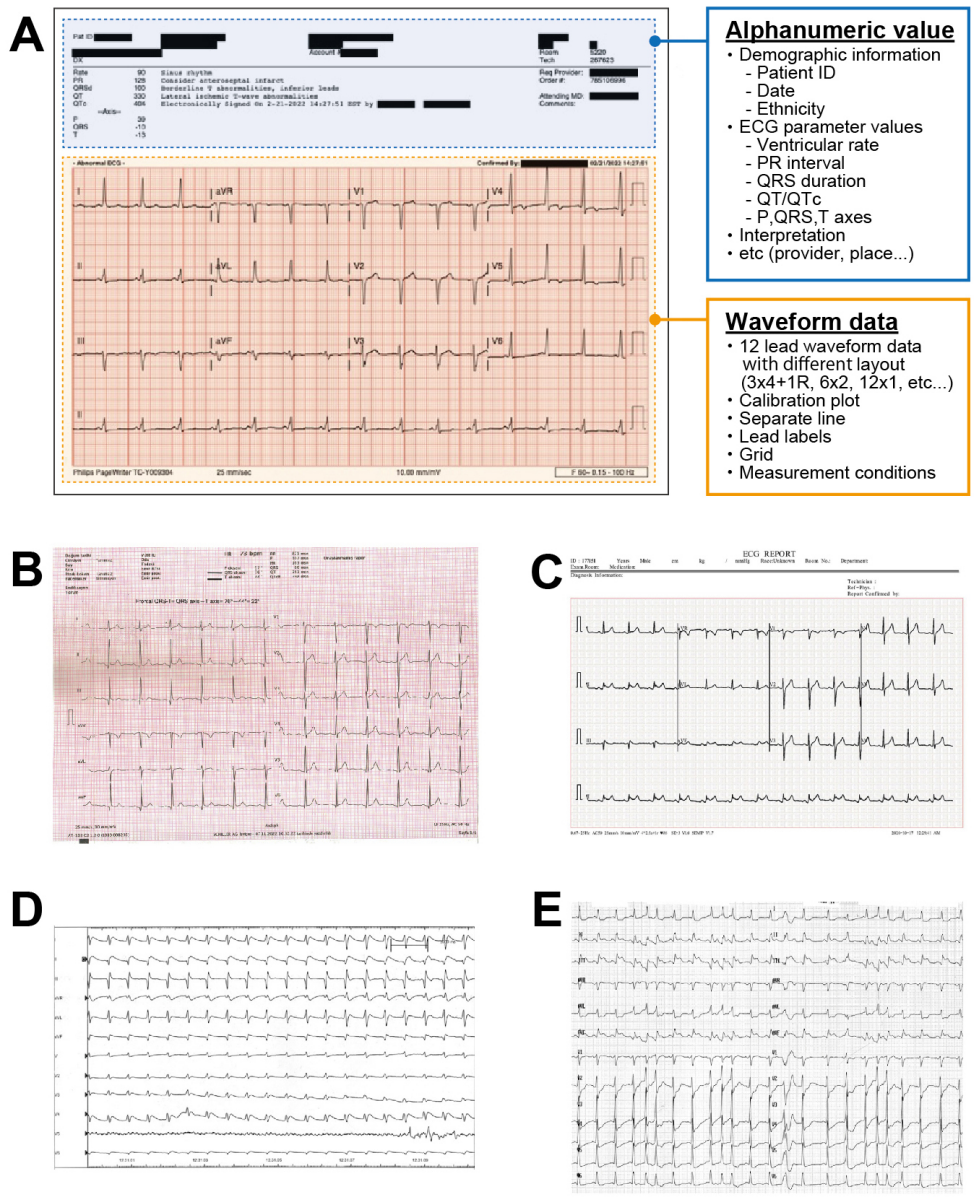
Schematic of ECG format variability ECG reports are typically recorded either on paper or as electronic PDF data. The ECG format includes various pieces of information, such as patient details, time, measurements, and automatic diagnostic results. One of the most significant differences in these formats is the table layout for displaying the 12 leads, which can be arranged in formats such as 3 × 4, 6 × 2, or 12 × 1. Additionally, the formats differ in the color or thickness of the grid, size, font, and location of captions, shape and size of calibration plots, and separation between adjacent leads. A. The ECG report is recorded as 3 × 4 + 1R with the calibration plots shown on the right. B. The ECG report is recorded on full grid paper as 6 × 2 with a single calibration plot. C. The ECG report is recorded as 3 × 4 + 1R with the calibration plots shown on the left. D. The ECG is recorded as 12 × 1 without the grid and calibration plot. E. The ECG is recorded as 12 × 2 with each of the 12 leads having two lead labels per waveform. ECG images are from references ^(60), (61), (62), (63)^.

The third problem lies in the accessibility of data for DL models, particularly for ECGs. Most modern ECG-DL models relied on raw voltage signals. Although signal recordings offer the advantages of small data sizes and minimal variation across devices and hospitals, medical professionals often lack access to raw ECG signals. This is caused by the attitude of manufacturers not making their voltage data openly available. They usually require a specialized software to extract them. For example, most large US vendors store their voltage data in a proprietary format and sell software to convert them to an openly readable format. In Japan, there has been an attempt to standardize the file format of ECG voltage recording with medical waveform format encoding rules (MFER) ^(55)^. However, vendors still use their proprietary format and provide software to convert to MFER, which incurs a substantial fee. Thus, accessibility problems persists. Consequently, conventional ECG images remain in high demand for daily diagnoses, leading to the development of DL models based on ECG images ^(56)^. Historically, ECG image-based DL models are not a groundbreaking concept ^(57), (58), (59)^. The proposed approaches were inspired by the workflow of clinicians who visually assessed ECG images. However, the image-based approach faces significant bottlenecks, particularly the difficulty in standardizing image data. There are various types of ECG formats across vendors and machines due to the lack of standardized regulations, making it infeasible for a single DL application to address all of them (Figure 5) ^(60), (61), (62), (63)^. Additionally, ECG image-based models may fail to accurately capture and focus on the voltage components of ECG images ^(62)^. If the model focused on areas outside the waveform region (i.e., patients background information often appearing above the waveform area), the generated prediction could be invalid. The model may also fail to correctly identify lead alignment, resulting in incorrect ECG interpretation. These fallacies may not be discernible to human observers. To establish a reliable DL pipeline, further research is required to elucidate the validity of the model prediction.

The fourth problem is related to cybersecurity. DL systems are particularly vulnerable to artificial noise, such as adversarial attacks. Adversarial changes to the original data are typically imperceptible to the human eye; however, they can be disruptive enough to cause DL models to misclassify samples ^(64)^. For example, adding an imperceptibly small vector ^(65)^ or a one-pixel attack ^(66)^ to the input data may result in incorrect predictions.

The advancement of DL along with the more general concept of applying high-performance computing (HPC) to the medical field not only is limited to direct clinical applications but also has shown utility in basic biological science ^(67)^. For example, the integration of DL with genome-wide association studies, which aim to uncover statistical associations between genetic variants and health-related traits in populations, is in its developmental phase. There is an emerging use of DL algorithm in a “reverse direction,” applied to phenotypes with multilateral modalities, such as cardiac age ^(68)^, whole human blood ^(69)^, and retinal fundus images ^(70)^, to detect hidden genetic changes. Moreover, the combination of DL with protein structural analysis accelerates protein analysis, such as molecular dynamics simulations, with the help of HPC techniques ^(71)^. These advances may facilitate the prediction of mechanisms underlying dynamic protein interactions affected by point mutations ^(72), (73)^ and large dynamic protein complex systems ^(74)^. These studies leveraging DL-enabled unprecedented technologies could play pivotal roles in advancing our understanding of diseases and basic biology in conjugation with big clinical data from a new perspective.

## 5. Conclusion

We reviewed the current status of DL application on ECG and echocardiogram in the cardiovascular field. DL models represent a promising technology that not only improves clinical workflows but may also serve as a useful tool in biological studies. Although several challenges remain, the values generated by the latest techniques are substantial. We expect that DL models will be incorporated into clinical practice and contribute to the health and well-being of patients in the near future. Additionally, we clearly foresee DL models playing important roles in understanding CVD biology.

## Article Information

### Conflicts of Interest

None

### Author Contributions

Masamitsu Nakayama and Ryuichiro Yagi contributed equally to this work.

## References

[1] James SL, Abate D, Abate KH, et al. Global, regional, and national incidence, prevalence, and years lived with disability for 354 diseases and injuries for 195 countries and territories, 1990-2017: a systematic analysis for the global burden of disease study 2017. Lancet. 2018;392(10159):1789-858. 30496104 10.1016/S0140-6736(18)32279-7 PMC6227754

[2] Tsao CW, Aday AW, Almarzooq ZI, et al. Heart disease and stroke statistics-2022 update: A report from the American Heart Association. Circulation. 2022;145(8):e153-639. 35078371 10.1161/CIR.0000000000001052

[3] Savarese G, Lund LH. Global public health burden of heart failure. Card Fail Rev. 2017;3(1):7-11. 28785469 10.15420/cfr.2016:25:2 PMC5494150

[4] Akhter Y, Singh R, Vatsa M. AI-based radiodiagnosis using chest X-rays: a review. Front Big Data. 2023;6:1120989. 37091458 10.3389/fdata.2023.1120989 PMC10116151

[5] Cheng PM, Montagnon E, Yamashita R, et al. Deep learning: an update for radiologists. RadioGraphics. 2021;41(5):1427-45. 34469211 10.1148/rg.2021200210

[6] Najjar R. Redefining radiology: a review of artificial intelligence integration in medical imaging. Diagnostics (Basel). 2023;13(17):2760. 37685300 10.3390/diagnostics13172760 PMC10487271

[7] Armoundas AA, Narayan SM, Arnett DK, et al. Use of artificial intelligence in improving outcomes in heart disease: A scientific statement from the American Heart Association. Circulation. 2024;149(14):e1028-50. 38415358 10.1161/CIR.0000000000001201 PMC11042786

[8] Goto S, Goto S. Application of neural networks to 12-lead electrocardiography ― current status and future directions. Circ Rep. 2019;1(11):481-6. 33693089 10.1253/circrep.CR-19-0096 PMC7897559

[9] Prineas RJ, Crow RS, Zhang ZM. The Minnesota code manual of electrocardiographic findings. New York: Springer Science & Business Media; 2009.

[10] Guglin ME, Thatai D. Common errors in computer electrocardiogram interpretation. Int J Cardiol. 2006;106(2):232-7. 16321696 10.1016/j.ijcard.2005.02.007

[11] Ribeiro AH, Ribeiro MH, Paixão GMM, et al. Automatic diagnosis of the 12-lead ECG using a deep neural network. Nat Commun. 2020;11(1):1760. 32273514 10.1038/s41467-020-15432-4 PMC7145824

[12] Zhu H, Cheng C, Yin H, et al. Automatic multilabel electrocardiogram diagnosis of heart rhythm or conduction abnormalities with deep learning: a cohort study. Lancet Digit Health. 2020;2(7):e348-57. 33328094 10.1016/S2589-7500(20)30107-2

[13] Cook DA, Oh SY, Pusic MV. Accuracy of physicians’ electrocardiogram interpretations: a systematic review and meta-analysis. JAMA Intern Med. 2020;180(11):1461-71. 32986084 10.1001/jamainternmed.2020.3989 PMC7522782

[14] Goto S, Kimura M, Katsumata Y, et al. Artificial intelligence to predict needs for urgent revascularization from 12-leads electrocardiography in emergency patients. PLOS ONE. 2019;14(1):e0210103. 30625197 10.1371/journal.pone.0210103 PMC6326503

[15] Attia ZI, Kapa S, Lopez-Jimenez F, et al. Screening for cardiac contractile dysfunction using an artificial intelligence–enabled electrocardiogram. Nat Med. 2019;25(1):70-4. 30617318 10.1038/s41591-018-0240-2

[16] Attia ZI, Noseworthy PA, Lopez-Jimenez F, et al. An artificial intelligence-enabled ECG algorithm for the identification of patients with atrial fibrillation during sinus rhythm: a retrospective analysis of outcome prediction. Lancet. 2019;394(10201):861-7. 31378392 10.1016/S0140-6736(19)31721-0

[17] Al-Zaiti SS, Martin-Gill C, Zègre-Hemsey JK, et al. Machine learning for ECG diagnosis and risk stratification of occlusion myocardial infarction. Nat Med. 2023;29(7):1804-13. 37386246 10.1038/s41591-023-02396-3 PMC10353937

[18] Cohen-Shelly M, Attia ZI, Friedman PA, et al. Electrocardiogram screening for aortic valve stenosis using artificial intelligence. Eur Heart J. 2021;42(30):2885-96. 33748852 10.1093/eurheartj/ehab153

[19] Kwon JM, Lee SY, Jeon KH, et al. Deep learning–based algorithm for detecting aortic stenosis using electrocardiography. J Am Heart Assoc. 2020;9(7):e014717. 32200712 10.1161/JAHA.119.014717 PMC7428650

[20] Galloway CD, Valys AV, Shreibati JB, et al. Development and validation of a deep-learning model to screen for hyperkalemia from the electrocardiogram. JAMA Cardiol. 2019;4(5):428-36. 30942845 10.1001/jamacardio.2019.0640 PMC6537816

[21] Lin C, Chau T, Lin CS, et al. Point-of-care artificial intelligence-enabled ECG for dyskalemia: a retrospective cohort analysis for accuracy and outcome prediction. npj Digit Med. 2022;5(1):8. 35046489 10.1038/s41746-021-00550-0 PMC8770475

[22] Liu CM, Shih ESC, Chen JY, et al. Artificial intelligence-enabled electrocardiogram improves the diagnosis and prediction of mortality in patients with pulmonary hypertension. JACC Asia. 2022;2(3):258-70. 36338407 10.1016/j.jacasi.2022.02.008 PMC9627911

[23] DuBrock HM, Wagner TE, Carlson K, et al. An electrocardiogram-based AI algorithm for early detection of pulmonary hypertension. Eur Respir J. 2024;64(1):2400192. 38936966 10.1183/13993003.00192-2024 PMC11269769

[24] Goto S, Mahara K, Beussink-Nelson L, et al. Artificial intelligence-enabled fully automated detection of cardiac amyloidosis using electrocardiograms and echocardiograms. Nat Commun. 2021;12(1):2726. 33976142 10.1038/s41467-021-22877-8 PMC8113484

[25] Goto S, Solanki D, John JE, et al. Multinational federated learning approach to train ECG and echocardiogram models for hypertrophic cardiomyopathy detection. Circulation. 2022;146(10):755-69. 35916132 10.1161/CIRCULATIONAHA.121.058696 PMC9439630

[26] Miura K, Yagi R, Miyama H, et al. Deep learning-based model detects atrial septal defects from electrocardiography: a cross-sectional multicenter hospital-based study. EClinicalmedicine. 2023;63:102141. 37753448 10.1016/j.eclinm.2023.102141 PMC10518511

[27] Yagi R, Goto S, Katsumata Y, et al. Importance of external validation and subgroup analysis of artificial intelligence in the detection of low ejection fraction from electrocardiograms. Eur Heart J Digit Health. 2022;3(4):654-7. 36710903 10.1093/ehjdh/ztac065 PMC9779862

[28] Noseworthy PA, Attia ZI, Behnken EM, et al. Artificial intelligence-guided screening for atrial fibrillation using electrocardiogram during sinus rhythm: a prospective non-randomised interventional trial. Lancet. 2022;400(10359):1206-12. 36179758 10.1016/S0140-6736(22)01637-3

[29] Yao X, Rushlow DR, Inselman JW, et al. Artificial intelligence–enabled electrocardiograms for identification of patients with low ejection fraction: a pragmatic, randomized clinical trial. Nat Med. 2021;27(5):815-9. 33958795 10.1038/s41591-021-01335-4

[30] Attia ZI, Friedman PA, Noseworthy PA, et al. Age and sex estimation using artificial intelligence from standard 12-lead ECGs. Circ Arrhythm Electrophysiol. 2019;12(9):e007284. 31450977 10.1161/CIRCEP.119.007284 PMC7661045

[31] Lima EM, Ribeiro AH, Paixão GMM, et al. Deep neural network-estimated electrocardiographic age as a mortality predictor. Nat Commun. 2021;12(1):5117. 34433816 10.1038/s41467-021-25351-7 PMC8387361

[32] Ladejobi AO, Medina-Inojosa JR, Shelly Cohen M, et al. The 12-lead electrocardiogram as a biomarker of biological age. Eur Heart J Digit Health. 2021;2(3):379-89. 36713596 10.1093/ehjdh/ztab043 PMC9707884

[33] Ouyang D, Theurer J, Stein NR, et al. Electrocardiographic deep learning for predicting post-procedural mortality: a model development and validation study. Lancet Digit Health. 2024;6(1):e70-8. 38065778 10.1016/S2589-7500(23)00220-0 PMC13184298

[34] Raghunath S, Ulloa Cerna AEU, Jing L, et al. Prediction of mortality from 12-lead electrocardiogram voltage data using a deep neural network. Nat Med. 2020;26(6):886-91. 32393799 10.1038/s41591-020-0870-z

[35] Lin CS, Liu WT, Tsai DJ, et al. AI-enabled electrocardiography alert intervention and all-cause mortality: a pragmatic randomized clinical trial. Nat Med. 2024;30(5):1461-70. 38684860 10.1038/s41591-024-02961-4

[36] Yagi R, Goto S, Himeno Y, et al. Artificial intelligence-enabled prediction of chemotherapy-induced cardiotoxicity from baseline electrocardiograms. Nat Commun. 2024;15(1):2536. 38514629 10.1038/s41467-024-45733-x PMC10957877

[37] Bunting KV, Steeds RP, Slater K, et al. A practical guide to assess the reproducibility of echocardiographic measurements. J Am Soc Echocardiogr. 2019;32(12):1505-15. 31653530 10.1016/j.echo.2019.08.015

[38] Gearhart A, Goto S, Deo RC, et al. An automated view classification model for pediatric echocardiography using artificial intelligence. J Am Soc Echocardiogr. 2022;35(12):1238-46. 36049595 10.1016/j.echo.2022.08.009 PMC9990955

[39] Knackstedt C, Bekkers SCAM, Schummers G, et al. Fully automated versus standard tracking of left ventricular ejection fraction and longitudinal strain: the FAST-EFs multicenter study. J Am Coll Cardiol. 2015;66(13):1456-66. 26403342 10.1016/j.jacc.2015.07.052

[40] Zhang J, Gajjala S, Agrawal P, et al. Fully automated echocardiogram interpretation in clinical practice. Circulation. 2018;138(16):1623-35. 30354459 10.1161/CIRCULATIONAHA.118.034338 PMC6200386

[41] Ouyang D, He B, Ghorbani A, et al. Video-based AI for beat-to-beat assessment of cardiac function. Nature. 2020;580(7802):252-6. 32269341 10.1038/s41586-020-2145-8 PMC8979576

[42] Reddy CD, Lopez L, Ouyang D, et al. Video-based deep learning for automated assessment of left ventricular ejection fraction in pediatric patients. J Am Soc Echocardiogr. 2023;36(5):482-9. 36754100 10.1016/j.echo.2023.01.015

[43] Duffy G, Cheng PP, Yuan N, et al. High-throughput precision phenotyping of left ventricular hypertrophy with cardiovascular deep learning. JAMA Cardiol. 2022;7(4):386-95. 35195663 10.1001/jamacardio.2021.6059 PMC9008505

[44] Deng Y, Cai P, Zhang L, et al. Myocardial strain analysis of echocardiography based on deep learning. Front Cardiovasc Med. 2022;9:1067760. 36588559 10.3389/fcvm.2022.1067760 PMC9800889

[45] He B, Kwan AC, Cho JH, et al. Blinded, randomized trial of sonographer versus AI cardiac function assessment. Nature. 2023;616(7957):520-4. 37020027 10.1038/s41586-023-05947-3 PMC10115627

[46] Holste G, Oikonomou EK, Mortazavi BJ, et al. Severe aortic stenosis detection by deep learning applied to echocardiography. Eur Heart J. 2023;44(43):4592-604. 37611002 10.1093/eurheartj/ehad456 PMC11004929

[47] Oikonomou EK, Holste G, Yuan N, et al. A multimodal video-based AI biomarker for aortic stenosis development and progression. JAMA Cardiol. 2024;9(6):534-44. 38581644 10.1001/jamacardio.2024.0595 PMC10999005

[48] Yuan N, Kwan AC, Duffy G, et al. Prediction of coronary artery calcium using deep learning of echocardiograms. J Am Soc Echocardiogr. 2023;36(5):474-81.e3. 36566995 10.1016/j.echo.2022.12.014 PMC10164107

[49] Valsaraj A, Kalmady SV, Sharma V, et al. Development and validation of echocardiography-based machine-learning models to predict mortality. EBiomedicine. 2023;90:104479. 36857967 10.1016/j.ebiom.2023.104479 PMC10006431

[50] Christensen M, Vukadinovic M, Yuan N, et al. Vision–language foundation model for echocardiogram interpretation. Nat Med. 2024;30(5):1481-8. 38689062 10.1038/s41591-024-02959-y PMC11108770

[51] Hata E, Seo C, Nakayama M, et al. Classification of aortic stenosis using ECG by deep learning and its analysis using grad-CAM. In2020 42nd Annual International Conference of the IEEE Engineering in Medicine & Biology Society (EMBC) 2020 Jul 20 (pp. 1548-51). IEEE. 10.1109/EMBC44109.2020.9175151 33018287

[52] Attia IZ, Tseng AS, Benavente ED, et al. External validation of a deep learning electrocardiogram algorithm to detect ventricular dysfunction. Int J Cardiol. 2021;329:130-5. 33400971 10.1016/j.ijcard.2020.12.065 PMC7955278

[53] König S, Hohenstein S, Nitsche A, et al. Artificial intelligence-based identification of left ventricular systolic dysfunction from 12-lead electrocardiograms: external validation and advanced application of an existing model. Eur Heart J Digit Health. 2024;5(2):144-51. 38505486 10.1093/ehjdh/ztad081 PMC10944686

[54] Yagi R, Mori Y, Goto S, et al. Routine electrocardiogram screening and cardiovascular disease events in adults. JAMA Intern Med. 2024;184(9). 10.1001/jamainternmed.2024.2270 PMC11217891 38949831

[55] Tamura K, Hirai M, Matsumoto K, et al. Medical waveform format encoding rules. Jpn J Electrocardiol. 2005;25(2):151-62.

[56] Sangha V, Mortazavi BJ, Haimovich AD, et al. Automated multilabel diagnosis on electrocardiographic images and signals. Nat Commun. 2022;13(1):1583. 35332137 10.1038/s41467-022-29153-3 PMC8948243

[57] Hedén B, Ohlsson M, Holst H, et al. Detection of frequently overlooked electrocardiographic lead reversals using artificial neural networks. Am J Cardiol. 1996;78(5):600-4. 8806356 10.1016/s0002-9149(96)00377-3

[58] Mori H, Inai K, Sugiyama H, et al. Diagnosing atrial septal defect from electrocardiogram with deep learning. Pediatr Cardiol. 2021;42(6):1379-87. 33907875 10.1007/s00246-021-02622-0

[59] Syeda-Mahmood T, Beymer D, Wang F. Shape-based matching of ECG recordings. In 2007 29th Annual International Conference of the IEEE Engineering in Medicine and Biology Society 2007 Aug 22 (pp. 2012-8). IEEE. 10.1109/IEMBS.2007.4352714 18002380

[60] Nguyen T, Pham HH, Le KH, et al. Detecting COVID-19 from digitized ECG printouts using 1D convolutional neural networks. PLOS ONE. 2022;17(11):e0277081. 36331942 10.1371/journal.pone.0277081 PMC9635737

[61] Sassi R, Sparagino L, Stockbridge NL, et al. Proof of concept for an international long-time preservation ECG format. Computing in Cardiology 2014, Cambridge, MA, USA, 2014, pp. 461-4.

[62] Sangha V, Nargesi AA, Dhingra LS, et al. Detection of left ventricular systolic dysfunction from electrocardiographic images. Circulation. 2023;148(9):765-77. 37489538 10.1161/CIRCULATIONAHA.122.062646 PMC10982757

[63] Romanò M. Text atlas of practical electrocardiography, A basic guide to ECG interpretation. Berlin: Springer; 2015. pp. 147-99.

[64] Han X, Hu Y, Foschini L, et al. Deep learning models for electrocardiograms are susceptible to adversarial attack. Nat Med. 2020;26(3):360-3. 32152582 10.1038/s41591-020-0791-x PMC8096552

[65] Goodfellow IJ, Shlens J, Szegedy C. Explaining and harnessing adversarial examples. arXiv preprint arXiv:1412.6572. 2014.

[66] Su J, Vargas DV, Sakurai K. One pixel attack for fooling deep neural networks. IEEE Trans Evol Computat. 2019;23(5):828-41.

[67] Goto S, McGuire DK, Goto S. The future role of high-performance computing in cardiovascular medicine and science -Impact of multi-dimensional data analysis. J Atheroscler Thromb. 2022;29(5):559-62. 34602525 10.5551/jat.RV17062 PMC9135644

[68] Shah M, de A Inácio MH, Lu C, et al. Environmental and genetic predictors of human cardiovascular ageing. Nat Commun. 2023;14(1):4941. 37604819 10.1038/s41467-023-40566-6 PMC10442405

[69] Homilius M, Zhu W, Eddy SS, et al. Perturbational phenotyping of human blood cells reveals genetically determined latent traits associated with subsets of common diseases. Nat Genet. 2024;56(1):37-50. 38049662 10.1038/s41588-023-01600-x PMC10786715

[70] Zekavat SM, Raghu VK, Trinder M, et al. Deep learning of the retina enables Phenome- and genome-wide analyses of the microvasculature. Circulation. 2022;145(2):134-50. 34743558 10.1161/CIRCULATIONAHA.121.057709 PMC8746912

[71] Abramson J, Adler J, Dunger J, et al. Accurate structure prediction of biomolecular interactions with AlphaFold 3. Nature. 2024;630(8016):493-500. 38718835 10.1038/s41586-024-07487-w PMC11168924

[72] Nakayama M, Goto S, Goto S. Physical characteristics of von Willebrand factor binding with platelet glycoprotein Ibɑ mutants at residue 233 causing various biological functions. TH Open. 2022;6(4):e421-8. 36632284 10.1055/a-1937-9940 PMC9729063

[73] Goto S, Oka H, Ayabe K, et al. Prediction of binding characteristics between von Willebrand factor and platelet glycoprotein Ibα with various mutations by molecular dynamic simulation. Thromb Res. 2019;184:129-35. 31739151 10.1016/j.thromres.2019.10.022

[74] Nakayama M, Goto S, Goto S. Development of the integrated computer simulation model of the intracellular, transmembrane, and extracellular domain of platelet integrin α IIb β 3 (platelet membrane glycoprotein: GPIIb–IIIa). TH Open. 2024;8(1):e96-105. 38425453 10.1055/a-2247-9438 PMC10904213

